# The Bacterial Biofilms: Formation, Impacts, and Possible Management Targets in the Healthcare System

**DOI:** 10.1155/cjid/1542576

**Published:** 2024-12-16

**Authors:** Eric Omori Omwenga, Silas Onyango Awuor

**Affiliations:** ^1^Department of Medical Microbiology & Parasitology, School of Health Sciences, Kisii University, Kisii, Kenya; ^2^Department of Applied Health Sciences, School of Health Sciences, Kisii University, Kisii, Kenya; ^3^Department of Medical Microbiology, Jaramogi Oginga Odinga Teaching and Referral Hospital, Kisumu, Kenya

**Keywords:** antimicrobial photodynamic therapy, antimicrobial resistance, bacterial biofilms, nanotechnology, natural compounds, phages

## Abstract

**Introduction:** The persistent increase in multidrug-resistant pathogens has catalyzed the creation of novel strategies to address antivirulence and anti-infective elements. Such methodologies aim to diminish the selective pressure exerted on bacterial populations, decreasing the likelihood of resistance emergence. This review explores the role of biofilm formation as a significant virulence factor and its impact on the development of antimicrobial resistance (AMR).

**Case Presentation:** The ability of bacteria to form a superstructure—biofilm—has made resistance cases in the microbial world a big concern to public health and other sectors as it is a crucial virulence factor that causes difficulties in the management of infections, hence enhancing chronic infection occurrence. Biofilm formation dates to about 3.4 billion years when prokaryotes were discovered to be forming them and since then due to evolution and growth in science, they are more understood.

**Management and Outcome:** The unique microenvironments within bacterial biofilms diminish antibiotic effectiveness and help bacteria evade the host immune system. Biofilm production is a widespread capability among diverse bacterial species. Biofilm formation is enhanced by quorum sensing (QS), reduction of nutrients, or harsh environments for the bacteria.

**Conclusion:** The rise of severe, treatment-resistant biofilm infections poses major challenges in medicine and agriculture, yet much about how these biofilms form remains unknown.

**Summary**

• What this study adds
• Emerging techniques like nanobiotechnology, phages, and CRISPR offer promising solutions to target bacteria more precisely, potentially reducing selective pressure and slowing the development of bacterial resistance.

## 1. Introduction

Biofilms are complex, surface-associated communities of microorganisms that play a crucial role in microbial survival and adaptability across diverse environments [1]. These microbial assemblies, formed by both Archaea and Eubacteria, date back over 3 billion years, with early evidence found in ancient geological formations in South Africa and Australia [2–5]. Biofilms have allowed microorganisms to thrive under extreme conditions on primitive Earth, and their evolutionary persistence highlights the advantages conferred by this lifestyle [1, 5]. Structured by both genetic and environmental factors, biofilms provide microbes with enhanced resistance to physical and chemical stresses, including temperature fluctuations, UV radiation, and nutrient scarcity [6–8]. This adaptability makes biofilms a prevalent and resilient form of microbial life, constituting an estimated 40%–80% of bacterial communities worldwide [9].

Despite their inherent importance, biofilms pose a major global health concern due to their ability to survive antitoxins and avoid developing resistance reactions. This adaptability contributes to persistent, challenging-to-treat conditions in various parts of the human body, such as the heart valves, lungs, urinary tract, and on medical devices like catheters and inserts [10–14]. It is crucial to comprehend the elements that contribute to biofilm formation and persistence since these microbial networks play a significant role in ecological cycles but can contribute to persistent health issues. Insights from biofilm research could lead to more effective strategies for managing their impacts, particularly in medical and industrial settings as in Table 1.

Although bacterial biofilms are often associated with negative impacts, particularly in healthcare and industrial settings, they also provide substantial benefits, especially in agriculture and environmental management. In agriculture, biofilms have proven valuable in delivering biocontrol agents to combat plant pathogens and in administering bio-fertilizers, both of which significantly boost crop productivity [25, 26]. Biofilms also play a crucial role in bioremediation, helping to degrade pollutants and restore contaminated environments, and in wastewater treatment, where they assist in purifying water by breaking down organic waste [27, 28]. Despite these advantages, the majority of biofilm research has focused on their harmful aspects, often overlooking their positive potential. By expanding our understanding of biofilms' beneficial roles, we can unlock new ways to utilize these microbial communities to enhance sustainable agriculture, promote environmental health, and improve overall human welfare in the years ahead.

## 2. Bacterial Biofilm Formation

**Figure 1:**
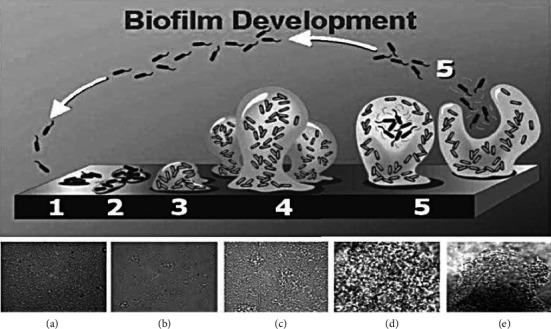
Diagram showing the development of a biofilm as a five-stage process. Stage 1: initial attachment of cells to the surface. Stage 2: extracellular polymeric substance (EPS) production, resulting in more firmly adhered “irreversible” attachment. Stage 3: early development of biofilm architecture. Stage 4: maturation of biofilm architecture. Stage 5: dispersion of single cells in the biofilm. The bottom panels (a–e) show the five stages of development represented by a photomicrograph of *P. aeruginosa* when grown under continuous-flow conditions on a glass substratum [1].

Bacteria frequently develop biofilms as a means of survival when faced with environmental challenges such as ultraviolet radiation, limited nutrients, extreme pH levels, high salinity, extreme temperatures, elevated pressure, and exposure to antimicrobial substances [23, 29, 30]. The process of biofilm formation occurs in multiple stages, encompassing attachment, maturation, and eventual detachment or dispersal. Initially, bacteria attach to surfaces, such as a tooth covered with a protein layer (pellicle), reversibly, during which they remain vulnerable to antibiotics—this vulnerability is crucial for the effectiveness of antibiotic prophylaxis in preventing infections during surgical procedures like alloplastic surgeries. As the biofilm progresses to maturity, bacteria adhere irreversibly to the surface, proliferate, and generate a polymeric matrix that envelops the colony. During this maturation phase, biofilms can attain significant thickness, sometimes reaching up to 50 *μ*m, and evolve into intricate three-dimensional structures resembling mushrooms or towers. The maturation process is shaped by various factors, including twitching motility, cell signaling, the production of extracellular polymeric substances (EPSs), and environmental conditions, all of which contribute to the biofilm's architecture [1]. Ultimately, certain areas of the biofilm may dissolve, releasing bacterial cells that can disperse and establish new colonies, facilitating the spread of biofilms. This dispersal can be instigated by elements such as bacteriophage activity within the biofilm [31–33]. Mature biofilms exhibit a high degree of organization, featuring water channels that enhance the transport of nutrients and waste, thereby functioning similarly to primitive multicellular organisms. Some motile bacteria utilize type IV pili to ascend to the upper layers of the biofilm, creating “hat-like” structures atop existing colonies [1, 34, 35]. This sophisticated organization of biofilms allows bacteria to flourish and adapt even in the most adverse environments as in Figure 1.

Biofilm development is a complex, multistage process that enables bacteria to form highly resilient communities on surfaces, leading to persistent and challenging infections, especially in medical environments. This process begins with bacterial cells initially attaching to a surface (Stage 1). Once attached, the bacteria produce a sticky EPS in Stage 2, anchoring them irreversibly. As they continue to grow and multiply, the biofilm's architecture begins to take shape in Stage 3. By Stage 4, the biofilm structure matures, becoming thicker and more robust, which enhances its resistance to environmental stressors. In the final Stage 5, individual bacterial cells disperse from the biofilm, spreading to new surfaces where the cycle can repeat. This multistep development of biofilms has been vividly demonstrated through photomicrographs of *Pseudomonas aeruginosa* biofilms grown on glass under continuous-flow conditions [1].

Biofilms, formed by various bacterial species, are a major cause of persistent infections, especially on medical devices like catheters, heart valves, and joint prostheses as in Table 2. The biofilm matrix protects bacteria from antibiotics and immune responses, making these infections particularly hard to treat. Biofilms on devices often lead to chronic urinary tract and bloodstream infections, while biofilms on tissues contribute to long-lasting infections, such as lung infections in cystic fibrosis, recurrent urinary tract infections, and chronic wounds. This resilience highlights the urgent need for innovative treatments that can effectively target and eliminate biofilm-associated infections in medical settings [36–38].

## 3. Bacterial Quorum Sensing (QS) and Biofilm Formation

Bacterial QS is a cell-to-cell communication process that enables bacteria to collectively modify their behavior in response to changes in the cell density and species composition of the surrounding microbial community. Such communication occurs in synchrony among bacteria species using small diffusible chemical signaling molecules called autoinducers (AIs) [39]. It has been documented to be responsible for the occurrence of various virulence traits among bacteria, among them being biofilms, toxins, and enzyme production among many [39]. Bacterial QS regulatory networks are not only very complicated but also vary among bacterial species [40]. This makes it hard to conclusively describe the regulatory mechanisms of QS on biofilm formation in general. However, based on the types of employed AIs, QS systems can be divided into several categories, namely, the *N*-acyl homoserine lactone (AHL) system that exists among Gram-negative bacteria. While the autoinducing peptide (AIP) system (known as AI-1 system previously) is found only in Gram-positive bacteria, AI-2 system and AI-3 system [41, 42] can be found in either.

**Figure 2:**
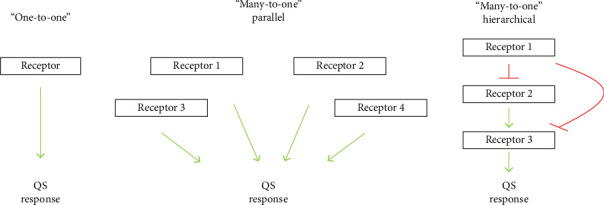
Illustration of three distinct configurations of quorum sensing (QS) networks utilized by bacteria for communication and the coordination of vital survival behaviors, including biofilm formation [43].

QS is mediated by diffusible signal molecules that reflect the density and physiological state of their population. Many pathogenic bacteria, such as *Staphylococcus aureus*, *Vibrio cholerae*, or *Pseudomonas aeruginosa*, regulate the expression of their virulence factors by QS. Such organisms use various networks or circuits to undertake their QS as shown in Figure 2 [39].

In the “One-to-One” model, a single receptor is responsible for detecting a specific signaling molecule, known as an AI, which subsequently governs the QS response. The “Many-to-One” parallel configuration enables the integration of signals from various AIs into a unified response pathway, facilitating a coordinated outcome. Conversely, the “Many-to-One” hierarchical configuration features multiple receptors arranged in a signaling cascade, where the activity of downstream receptors is influenced by those upstream, thereby introducing additional layers of regulatory control [39].

*Pseudomonas aeruginosa*, a Gram-negative bacterium, uses QS to regulate its biofilm formation and virulence. Through QS, *P. aeruginosa* cells produce signaling molecules—specifically OdDHL and BHL—via two key enzymes encoded by the genes *lasI* and *rhlI*. As these molecules accumulate, they bind to receptors LasR and RhlR, activating genes linked to virulence and biofilm development [44, 45]. The *rhl* system specifically regulates swarming motility, which aids in bacterial colonization, and the production of pyocyanin, a toxin that damages host tissues, enhancing infection potential [32, 39, 46].

**Figure 3:**
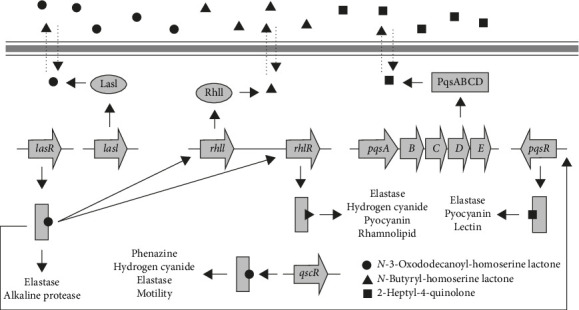
Quorum sensing control of gene expression in *P. aeruginosa* [39].

The *las* system in bacteria regulates genes that produce key molecules like elastase, alkaline protease, and endotoxin A, all of which are essential for biofilm maturation and bacterial virulence. These molecules not only strengthen biofilms but also enhance the bacteria's pathogenic potential. Working alongside the *rhl* system, the *las* system is part of a QS network, allowing bacteria to coordinate biofilm formation and virulence in response to their population density as in Figure 3. Together, these systems showcase the sophisticated regulatory mechanisms bacteria use to adapt and maintain infectious capabilities [43].

QS plays a critical role in bacterial communication and biofilm formation, allowing bacteria to regulate gene expression based on cell density through signaling molecules. In *Pseudomonas aeruginosa*, QS uses AHLs to coordinate biofilm development and other communal behaviors [33, 39, 47].

In *Escherichia coli*, while the bacteria lack an AHL-producing gene (analogous to the luxI gene in *P. aeruginosa*), they do have the receptor gene sdiA, similar to the luxR gene in *P. aeruginosa*. This receptor can detect AHLs produced by other bacterial species, enabling *E. coli* to respond by increasing biofilm-related processes, such as EPS production and cell attachment [48, 49].

In addition, many Gram-negative microscopic organisms, including *Vibrio cholerae*, *Vibrio parahaemolyticus*, and *E. coli*, rely on an additional flagging framework for biofilm arrangement due to the artificial intelligence 2 atom [48–51]. In *E. coli*, biofilm-related properties are initiated when intelligence 2 is phosphorylated after being transported into the cell via an ABC carrier [41, 54]. The LuxPQ receptor complex, which separates the man-made intelligence 2 particle in *V. cholerae*, modifies LuxQ activity to reduce biofilm growth by inhibiting biofilm-advancing quality record at high artificial intelligence 2 levels [54].

In contrast, Gram-positive bacteria primarily use AIPs as signaling molecules. In *Staphylococcus aureus*, the agr system, composed of genes like agrA, agrB, agrC, agrD, and hld, is crucial for the final dispersion phase of biofilm formation. Notably, agr mutants form thicker biofilms, likely because they are less able to detach from mature biofilms [55–57].

These insights into bacterial QS mechanisms highlight the importance of QS in biofilm formation across bacterial species. Understanding these pathways offers promising therapeutic strategies, such as QS antagonists that inhibit specific signaling steps, potentially aiding in the management and treatment of biofilm-associated infections [58–60].

## 4. Biofilms in the Food Industry

In the food industry, surfaces and equipment frequently become colonized by microorganisms that form biofilms—complex communities of bacteria encased in an extracellular matrix. This matrix, mainly composed of polysaccharides, proteins, and sometimes cellulose, allows bacteria to adhere strongly to surfaces such as stainless steel, wood, rubber, and even food products like meat and fruit [61, 62]. The matrix structure creates gradients that regulate oxygen and nutrient flow and contains enzymes that support bacterial survival, making biofilms exceptionally resilient against cleaning agents and disinfectants [63].

Pathogenic bacteria, including foodborne species, thrive in these environments, often forming multispecies biofilms. Some pathogens, like *Listeria monocytogenes*, are weak biofilm producers but can attach to surfaces by associating with stronger biofilm-forming bacteria such as *Enterococcus faecium* and *Enterococcus faecalis* [64]. Similarly, *E. coli* has shown enhanced biofilm formation when interacting with other strains like *Burkholderia caryophylli* and *Ralstonia insidiosa* found in food processing plants [62, 63].

Biofilm formation follows a structured process: First, surfaces are conditioned, and cells attach reversibly, which then becomes permanent as microcolonies develop. Over time, these colonies mature into a three-dimensional structure, creating a stable ecosystem that can disperse cells to new locations [65–67]. Biofilms adapt based on environmental conditions and bacterial species involved, which allows them to vary in structure and resilience. This variability, combined with the diverse materials in food processing, makes biofilm removal difficult [68].

## 5. Impact of Biofilm on the Food Industry

While biofilms are often associated with contamination, they also offer unique benefits in the food and agricultural industries. As Seixas, Moreira, and Kehrig [69] noted, the positive applications of biofilms are due to their strength, flexibility, water solubility, and permeability to water vapor. Biofilms can act as protective barriers, potentially preventing lipid oxidation in foods and reducing the transfer of gases, moisture, and odors [70].

In agriculture, biofilms enhance plant health and can boost crop productivity. In food processing, they play a significant role in wastewater treatment, aiding in the reduction of excess sludge and improving water quality. Additionally, biofilms have been harnessed to create edible films and biodegradable packaging, supporting environmentally friendly packaging solutions.

## 6. Resistance to Antibiotics in Biofilm Communities

Biofilms play a crucial role in bacterial antibiotic resistance, particularly in chronic infections. While antibiotics have been central to infection control for decades, bacteria have evolved resistance mechanisms, including biofilm formation, which protects them from both antibiotics and the immune system [71]. Biofilms are structured communities surrounded by a protective matrix that restricts antibiotic penetration and shields bacteria, leading to persistent infections with ongoing inflammation and tissue damage [72, 73].

In biofilms, bacterial resistance involves both tolerance (surviving without growth) and resistance (growing despite antibiotic presence). Within biofilms, zones of slow-growing or dormant cells arise due to nutrient limitations, metabolic gradients, and adaptive stress responses. These cells, often called “persister” cells, are highly tolerant to antibiotics, as they are not affected by drugs targeting active cellular functions [74, 75].

Biofilms resist antibiotics through multiple mechanisms: limited penetration due to the dense biofilm matrix, adaptive stress responses, and the development of persister cells. Additionally, physiological changes—such as slowed metabolic activity in deeper biofilm layers—further protect bacteria from antibiotics. In the case of staphylococcal biofilms, for example, cells in nutrient-limited areas enter a dormant state, reducing susceptibility to antibiotics that target growth processes [76–78].

This multifaceted resistance makes biofilm-related infections like cystic fibrosis and chronic pneumonia especially difficult to treat, emphasizing the need for new therapeutic approaches to effectively manage and disrupt biofilms.

## 7. Pathogen Mechanisms of Antibiotic Resistance

**Figure 4:**
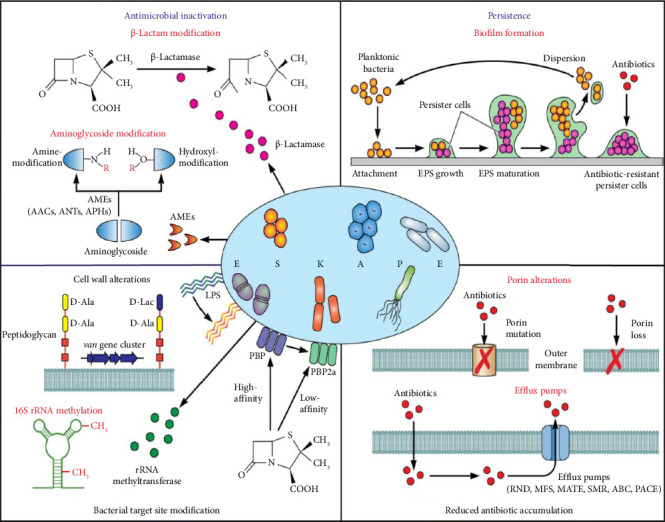
Mediators of antimicrobial resistance in ESKAPE pathogens [79].

In clinical settings, ESKAPE pathogens—*Enterococcus faecium, Staphylococcus aureus, Klebsiella pneumoniae, Acinetobacter baumannii, Pseudomonas aeruginosa*, and *Escherichia coli*—pose a significant threat due to their robust antibiotic resistance mechanisms. These pathogens have developed multiple survival strategies, making them exceptionally difficult to treat. One common mechanism is the production of enzymes that inactivate or modify antibiotics, rendering them ineffective. They may also alter their internal target sites, preventing antibiotics from binding and disrupting bacterial processes. Additionally, ESKAPE bacteria can restrict antibiotic penetration by modifying cell permeability or actively expelling drugs through efflux pumps, which lowers the drug concentration within the cell. Many of these bacteria can also form biofilms, complex communities that adhere to surfaces and act as protective barriers, enabling bacteria to persist even in the presence of antibiotics [79]. These combined strategies allow ESKAPE pathogens to evade standard treatments, making them a formidable challenge in healthcare and underscoring the urgent need for new antimicrobial solutions as in Figure 4.

Mechanisms that drive antimicrobial resistance (AMR) in ESKAPE pathogens can be grouped into four main categories:

1. Enzyme-mediated antimicrobial inactivation: This process involves enzymes that either irreversibly destroy the antibiotic's active site or modify structural elements of the drug, reducing its effectiveness. For example, *β*-lactamases hydrolyze the *β*-lactam ring, neutralizing the antibiotic, while aminoglycoside-modifying enzymes alter the antibiotic's hydroxyl or amino groups to inhibit its interaction with bacterial targets [79].
2. Modification of bacterial target sites: By altering antibiotic binding sites, bacteria can prevent or reduce the binding affinity of antibiotics. This includes surface-level modifications, such as lipopolysaccharide (LPS) alterations and penicillin-binding protein PBP2a expression, which lowers *β*-lactam binding affinity, as well as intracellular modifications like 16S rRNA methylation. Genes like the van gene cluster contribute to peptidoglycan alterations that further inhibit antibiotic action [79].
3. Reduced antibiotic accumulation: Bacteria have developed effective ways to resist antibiotics by lowering the drugs' concentration within their cells. One strategy involves modifying or removing outer membrane channels, or porins, such as OprD in *Pseudomonas aeruginosa*, CarO in *Acinetobacter baumannii*, and OmpK36 in *Klebsiella pneumoniae*, to prevent antibiotics from entering. Additionally, bacteria use efflux pumps—systems like RND, MFS, MATE, SMR, ABC, and PACE—to actively expel antibiotics from the cell, keeping their intracellular levels low. These combined defenses make bacteria more resilient to antibiotics and complicate treatment [79].
4. Persistence through biofilm formation: Bacteria within biofilms are far more resistant to antibiotics than free-floating cells, thanks to the EPS matrix that surrounds and protects them, allowing infections to persist longer and resist treatment [79].

## 8. Role of Bacterial Biofilms in AMR

Biofilms play a critical role in AMR due to their highly complex structure. Compared to free-floating (planktonic) bacteria, the same bacteria in biofilms can show a 10- to 1000-fold increase in resistance to antibiotics [80, 81]. For instance, studies on *Staphylococcus epidermidis* showed that while 100% of planktonic isolates were susceptible to vancomycin, nearly 75% were completely resistant to the same antibiotic when in biofilm form [39, 82]. *Klebsiella pneumoniae* demonstrates a similar pattern, appearing susceptible in liquid tests but becoming highly resistant within biofilms [82].

While common resistance mechanisms—such as point mutations, enzymes, and efflux pumps—contribute to AMR, they do not fully explain the heightened resistance in biofilms [72, 83]. Within biofilms, multiple factors work together to reduce or block antibiotic activity, allowing bacteria to survive even in high antibiotic concentrations, a phenomenon known as recalcitrance [84].

There are three primary mechanisms by which biofilms enhance bacterial resistance to antibiotics:

1. Resistance at the biofilm surface: The biofilm's outer layers create a sticky, slimy matrix composed of exopolysaccharides, DNA, and proteins, which makes it challenging for antibiotics to penetrate and reach the bacteria within. This structure slows the diffusion of antibiotics, often allowing them to be inactivated before reaching their targets. However, the effectiveness of this surface-level resistance can vary depending on the biofilm [85].
2. Resistance within biofilm microenvironments: If antibiotics penetrate the biofilm's surface, they encounter an inhospitable microenvironment deeper within. Here, metabolic byproducts, nutrient gradients, and oxygen depletion create anaerobic zones that impact antibiotic efficacy. For example, low oxygen levels reduce the bactericidal effects of antibiotics like tobramycin and ciprofloxacin, while pH changes impair the action of aminoglycosides [81].
3. Resistance of bacterial “persister” cells: Deep inside the biofilm, some bacterial cells adopt a dormant, “spore-like” state, making them highly resistant to antibiotic treatment. These “persister” cells do not divide and remain dormant, evading antibiotics. Notably, their survival is not due to genetic mutations, as they return to normal susceptibility once they re-emerge from dormancy and begin to divide [80].

**Figure 5:**
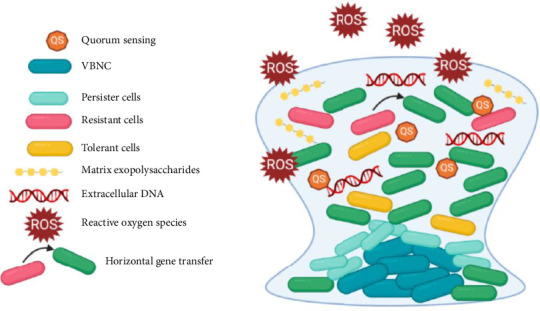
Mechanisms leading to resistance occur simultaneously within a mature biofilm.

Biofilms offer additional advantages for bacteria, particularly by enabling proximity between multiple organisms. This proximity allows for bacterial communication through QS [86] and supports the transfer of mobile genetic elements, such as plasmids, which enhances the spread of resistance traits. The biofilm environment not only stabilizes plasmids but also facilitates the transmission of DNA elements that encode biofilm-promoting factors, further reinforcing the biofilm's resilience and sustaining infections in patients [87] as described in Figure 5.

## 9. Approaches in the Control of Bacterial Biofilms and Related Infections

Natural products have been found to offer an alternative in the management of biofilms and hence could be a key strategy in the control of ailments that affect humankind. Various sites can be targeted in managing them. Since QS has been found to play a key role in the formation and dispersal of biofilms, it could be one of those pathways that can be targeted. The compounds that can inhibit such a circuit may offer antagonistic activity by inhibiting production, release, or AI detection, hence controlling biofilm formation. Several compounds have been detected especially from natural products that can assist and they include but are not limited to essential oils [88], wheat bran [89], garlic [90], cinnamon [91], furanones, and oroidin [91], among many more.

A promising approach to blocking biofilm formation is to disrupt the production of cyclic di-GMP (c-di-GMP), a key bacterial signaling molecule that regulates growth, cell differentiation, and virulence. C-di-GMP is especially important for biofilm development, as it controls the production of adhesins and exopolysaccharides that help bacteria stick together. Found only in bacteria—not in eukaryotes or Archaea—c-di-GMP is regulated by two enzyme types: diguanylate cyclases (DGCs), which produce it, and phosphodiesterases (PDEs), which break it down, allowing bacteria to precisely manage essential biofilm-related processes [92].

**Figure 6:**
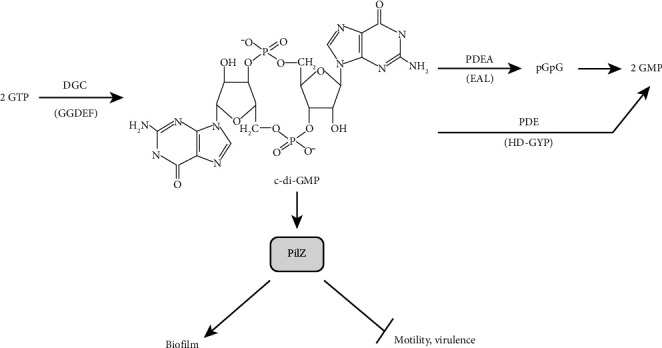
The c-di-GMP regulatory pathway [92].

Targeting this pathway offers a novel approach to biofilm control. For instance, deleting DGCs completely halts biofilm formation, as these enzymes are responsible for producing c-di-GMP, an essential step in biofilm development as in Figure 6.

c-di-GMP is a key regulatory molecule in bacteria that influences behaviors like biofilm formation and motility, helping bacteria adapt to their environment. When c-di-GMP levels are high, bacteria tend to form biofilms by producing sticky extracellular substances, anchoring them in place and enhancing resistance to stress. In contrast, low c-di-GMP levels favor bacterial movement and, in some cases, increase virulence. This balance is maintained by two types of enzymes: DGCs, which produce c-di-GMP, and PDEs, which degrade it. Proteins with PilZ domains detect c-di-GMP, but exactly how this molecule directs specific responses remains a subject of ongoing research [92].

**Figure 7:**
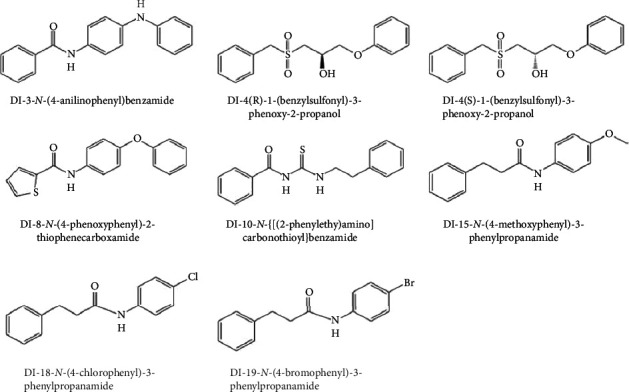
Chemical structures of the diguanylate cyclase (DGC) inhibitors [94].

A promising compound for targeting this pathway is N-[4-(phenylamino)phenyl]-benzamide, which has demonstrated broad-spectrum activity by decreasing biofilm formation in both *V. cholerae* and *P. aeruginosa* [93, 94]. Additionally, other compounds (Figure 7) have shown antagonistic effects against DGC without affecting microbial growth, meaning they do not impose selective pressure on bacteria and may lower the risk of developing resistance [94].

In addition to traditional antimicrobials, natural compounds that promote biofilm dispersal show potential for biofilm control, particularly those targeting the breakdown of the extracellular polysaccharides in the biofilm matrix. An example is norspermidine, which, when combined with silver nitrate, was shown to disperse multispecies wastewater biofilms effectively [95, 96].

The bioavailability and bioactivity of these natural compounds can be enhanced through nanoencapsulation, significantly improving their impact on biofilms. For instance, chitosan-encapsulated baicalein and quercetin flavonoids have been shown to inhibit biofilm formation on *E. coli* Top10 QS biosensor strains at much lower dosages compared to nonencapsulated flavonoids (Omwenga et al., 2017). In a study by Ellboudy et al. [98], a liposome-encapsulated cinnamon oil and colistin nanoformula (5.0 mg/mL each) exhibited excellent biofilm inhibition against *S. aureus*. Another example is chitosan/cyclodextrin nanocapsules loaded with naringenin, which inhibited biofilm formation in *E. coli* by up to 60%, illustrating the synergistic effect of positively charged chitosan with naringenin's bioactivity, leveraging the nanocapsule's high surface area-to-volume ratio [99]. These studies highlight the promising role of nanobiotechnology in advancing biofilm management.

Photodynamic therapy (PDT) is another innovative biofilm control strategy. PDT involves using photosensitizer (PS) molecules that bind to biofilm cells and, upon absorbing light of a specific wavelength, become excited to produce reactive oxygen species (ROS). These ROS cause oxidative damage, leading to cell death. PDT can follow different mechanisms: Type I, in which 3PS∗ transfers electrons or hydrogen atoms to oxygen to generate ROS, and Type II, in which 3PS∗ transfers energy to oxygen to produce ROS, while a Type III mechanism provides an oxygen-independent way to kill anaerobes [100]. In one study, PDT using a 660 nm laser and PSs like methylene blue and photoditazine showed a significant reduction in *Streptococcus mutans* biofilm, achieving up to a 6-log bacterial reduction [101]. Additionally, a red LED PDT treatment with a cationic nanoemulsion-coated chloro-aluminum phthalocyanine PS effectively treated oral candidiasis by reducing adhesion, biofilm formation, and virulence of *Candida albicans* [85].

CRISPR-Cas9 technology is also being explored for biofilm management by targeting specific genes that regulate biofilm formation and antibiotic resistance. CRISPR-Cas9 can edit genes on bacterial plasmids, potentially reversing AMR and inhibiting biofilm formation. Research includes preventing biofilm formation in *E. coli* [102], *S. mutans* [103], and *S. aureus* [104], while studies on *Pseudomonas fluorescens* showed that CRISPR interference (CRISPRi) of biofilm-associated genes reduced biofilm thickness and mass [105]. Another study targeted the *ompA* gene in *Cedecea neteri*, leading to reduced biofilm formation and lower infection rates [106]. These findings emphasize CRISPR's potential for precise genetic manipulation in managing biofilm-associated infections.

**Figure 8:**
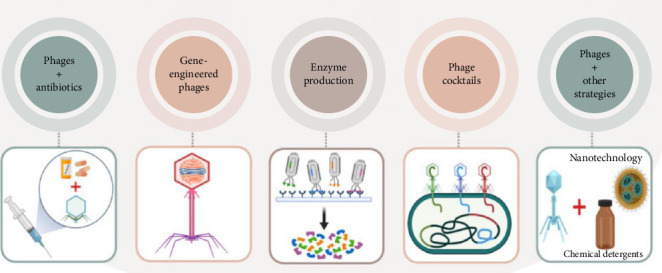
Future directions in phage therapy and its potential applications for the prevention and treatment of bacterial biofilms [110].

Bacteriophage (phage) therapy is another promising biofilm control strategy. Phages produce enzymes like depolymerases, holins, and endolysins that degrade biofilm matrices and disrupt bacterial cell walls, facilitating the penetration of phages into the biofilm's deeper layers [107, 108]. Depolymerases, for instance, recognize and digest EPS, weakening the biofilm structure [109], while endolysins, which cleave bacterial peptidoglycan, are released during phage infection to help disintegrate biofilm integrity. Phage therapy has unique advantages over antibiotics, including the ability to navigate through biofilm pores and channels, reaching and lysing bacteria within the biofilm. Future research could focus on enhancing phage stability, infectivity, and delivery by integrating phage therapy with nanobiotechnology, bioengineering phages, using phage cocktails, and combining phages with sublethal antibiotic doses for synergistic effects as in Figure 8 [111, 112].

## 10. Conclusion

Biofilm resilience against antibiotics is a major factor complicating the treatment of biofilm-associated infections. Significant progress has been made in understanding the factors that contribute to this resistance, including the role of persister cells and the molecular mechanisms that generate them. This has already led to the development of several promising antibiofilm treatment strategies.

Despite these advances, much about bacterial biofilm formation remains unexplored. The increasing prevalence of severe biofilm infections and their resistance to antimicrobial treatments poses substantial challenges not only in medicine but also in fields like agriculture. An effective approach to combat biofilm-related issues should involve the discovery of new antibiofilm molecules and targeted modifications in QS signaling pathways. This review covered biofilm formation and mechanisms of action for selected antibiofilm compounds. Understanding these mechanisms provides insights into biofilm characteristics that can inform the development of new drugs targeting known pathways, enhancing the effectiveness of existing therapies. Combination strategies, which pair less effective antibiotics with potent antibiofilm agents, could also increase antibiotic activity.

The use of cutting-edge technologies like nanobiotechnology, phages, and CRISPR is essential and shows promise, though more research is needed to establish standardized methods that minimize selective pressure on bacteria and reduce the risk of resistance. Validating these novel approaches will require closer integration of fundamental research with clinical applications to bring these techniques into therapeutic practice against challenging infections.

## Figures and Tables

**Table 1:** Biofilm-forming bacteria on medical devices.

Bacterial species	Associated medical devices	Reference
*Staphylococcus epidermidis*	Catheters, prosthetic joints, heart valves	[17, 18]
*Staphylococcus aureus*	Catheters, orthopedic implants, heart valves	[19]
*Pseudomonas aeruginosa*	Ventilators, catheters, urinary catheters	[20]
*Escherichia coli*	Urinary catheters	Murugan et al.,2016
*Klebsiella pneumoniae*	Endotracheal tubes, urinary catheters	[17]
*Enterococcus faecalis*	Catheters, heart valves	[21]
*Candida albicans* (fungal)	Urinary catheters, intravenous catheters	[22]; Sharme et al., 2023
*Acinetobacter baumannii*	Ventilators, catheters, wound dressings	[23]
*Proteus mirabilis*	Urinary catheters	[17]
*Serratia marcescens*	Respiratory and urinary catheters	[17]
*Streptococcus mutans*	Dental implants	[23]
*Burkholderia cepacia*	Cystic fibrosis respiratory equipment	[24]
*Mycobacterium avium*	Endoscopes, bronchoscopes	[23]

**Table 2:** A summary table showing the common human bacterial species infection/disease and attachment/surface (adopted from [36]).

Bacterial species	Infection/disease	Attachment site/surface
*Staphylococcus aureus*	Skin infections, pneumonia, sepsis	Skin, nasal passages, wounds
*Escherichia coli*	Urinary tract infections, gastroenteritis	Intestinal tract, urinary tract
Streptococcus *pneumoniae*	Pneumonia, meningitis, otitis media	Respiratory tract, middle ear
*Helicobacter pylori*	Gastritis, peptic ulcers	Stomach lining
*Pseudomonas aeruginosa*	Wound infections, respiratory infections	Skin, respiratory tract, wounds
*Neisseria gonorrhoeae*	Gonorrhea	Urogenital tract
*Clostridium difficile*	Colitis	Intestinal tract
*Mycobacterium tuberculosis*	Tuberculosis	Lungs (alveoli)
*Salmonella* species	Gastroenteritis, typhoid fever	Intestinal tract
*Streptococcus pyogenes*	Pharyngitis, skin infections	Throat, skin
*Haemophilus influenzae*	Respiratory infections, meningitis	Respiratory tract
*Klebsiella pneumoniae*	Pneumonia, urinary tract infections	Lungs, urinary tract

## Data Availability

All data relevant to the study are included within this article.
